# Tree Trunk Recognition in Orchard Autonomous Operations under Different Light Conditions Using a Thermal Camera and Faster R-CNN

**DOI:** 10.3390/s22052065

**Published:** 2022-03-07

**Authors:** Ailian Jiang, Ryozo Noguchi, Tofael Ahamed

**Affiliations:** 1 Graduate School of Science and Technology, University of Tsukuba, 1-1-1 Tennodai, Tsukuba 305-8577, Japan; s2026037@u.tsukuba.ac.jp; 2 Faculty of Life and Environmental Sciences, University of Tsukuba, 1-1-1 Tennodai, Tsukuba 305-8577, Japan; noguchi.ryozo.gm@u.tsukuba.ac.jp

**Keywords:** thermal image, tree trunk detection, low-light conditions, orchards, faster R-CNN

## Abstract

In an orchard automation process, a current challenge is to recognize natural landmarks and tree trunks to localize intelligent robots. To overcome low-light conditions and global navigation satellite system (GNSS) signal interruptions under a dense canopy, a thermal camera may be used to recognize tree trunks using a deep learning system. Therefore, the objective of this study was to use a thermal camera to detect tree trunks at different times of the day under low-light conditions using deep learning to allow robots to navigate. Thermal images were collected from the dense canopies of two types of orchards (conventional and joint training systems) under high-light (12–2 PM), low-light (5–6 PM), and no-light (7–8 PM) conditions in August and September 2021 (summertime) in Japan. The detection accuracy for a tree trunk was confirmed by the thermal camera, which observed an average error of 0.16 m for 5 m, 0.24 m for 15 m, and 0.3 m for 20 m distances under high-, low-, and no-light conditions, respectively, in different orientations of the thermal camera. Thermal imagery datasets were augmented to train, validate, and test using the Faster R-CNN deep learning model to detect tree trunks. A total of 12,876 images were used to train the model, 2318 images were used to validate the training process, and 1288 images were used to test the model. The mAP of the model was 0.8529 for validation and 0.8378 for the testing process. The average object detection time was 83 ms for images and 90 ms for videos with the thermal camera set at 11 FPS. The model was compared with the YOLO v3 with same number of datasets and training conditions. In the comparisons, Faster R-CNN achieved a higher accuracy than YOLO v3 in tree truck detection using the thermal camera. Therefore, the results showed that Faster R-CNN can be used to recognize objects using thermal images to enable robot navigation in orchards under different lighting conditions.

## 1. Introduction

Between 1995 and 2010, Japan’s agricultural labor force has gradually declined from 4.14 to 2.39 million, and its average age increased from 59.1 years to 65.8 years [1]. Therefore, agricultural robotics have the potential to support agricultural labor shortages and increase agricultural productivity in this critical stage of transformation [2]. In agricultural automation and robotics applications, vehicle navigation is important in outdoor environments, which are complex and uncertain compared to indoor conditions [3]. Open field navigation has had significant success using the Real Time Kinematic Global Navigation Satellite System (RTK-GNSS) with higher accuracy [4]. However, orchard navigation is the most complex, and interruption of RTK-GNSS signals due to high and dense canopies is frequently reported [5]. As Japanese orchards have net and dense branches, GNSS signals may be affected, and many farmland orchards do not have base stations set up to use GNSS directly. In addition, the performance of GNSS-based navigation depends highly on GNSS signal quality.

Therefore, orchard-based navigation systems remain a challenge for the development and application of agricultural robots. Light Detection and Ranging (LiDAR) is used to scan the surrounding environment in real time and returns accurate distance information using the time-of-flight principle. It can overcome the limitations of low light and interruption of RTK-GNSS signals. Laser sensors have been used for agricultural position detection and automatic coupling using artificial landmarks, concrete, and grass surface ground for navigation [6,7,8] or crop fields [9] by processing point clouds for crop and weed detection. However, LiDAR can only obtain distance and angular orientation information; it cannot provide information about the object’s type. To develop an intelligent robust system, LiDAR may not be sufficient for low-light conditions. Using a camera as a sensor is advantageous due to its low cost and ease of installation in agricultural robots. Camera sensors are widely used in navigation systems in various ways, such as using color information to segment paths in citrus groves for vehicle navigation [10], or focusing on color information of shadows and soil textures, to distinguish traversable areas (furrows) from impenetrable areas (ridges) for navigation [11]. However, common RGB cameras are easily affected by light, particularly in orchards, where the light differs between shaded and nonshaded areas, and the cameras typically experience exposure phenomena, making target identification unclear. Furthermore, low light is one of the most difficult aspects to create effective vision systems for agricultural robotics [12]. RGB cameras have high light requirements and work with low accuracy or are even unsuitable for low-light environments [13].

A thermal sensor measures the temperature of an object and the amount of heat it emits. Based on this imaging principle, a thermal camera was used to assess the health of tree trunks and detect damage in the interior [14]. Additionally, the thermal camera has a detection function that is not affected by visible light; thus, this study used a thermal camera to detect tree trunks in orchards for target detection. The target can be used to position a robot during navigation. In addition to thermal imagery, advancements in artificial intelligence and deep learning can be used for tree-trunk detection to enable vehicle navigation under different lighting conditions. Similar targets may have the same range of temperatures that emit and can easily localize the target using computer vision-based deep learning approaches [15]. Da Silva et al. compared the results of Yolo Only Look Once (YOLO) and Single Shot MultiBox Detector (SSD) for thermal images from forests, and the proposed use of thermal images allowed the execution of in-field forestry operations during the day and night [16].

In target detection, computer vision technology combines target localization and target classification; uses image processing, machine learning, and other techniques to identify the class of targets in the image; and returns the position of the target, generally using a rectangular box to indicate its position [17]. In general, the detection process is divided into two steps: (1) determine whether there is a target in the input image using target classification, and (2) perform labeling to search for the target using the bounding box [18]. This algorithm provides the accurate position of each target while accurately determining each target’s type. In the target-detection algorithm, images are stored as a pixel matrix, and the image features of the target class and position must be obtained before target detection [19]. The traditional method of machine learning is based on image processing, it lacks specificity for region selection, which typically causes sliding window redundancy and high time complexity, and in the process of dynamic change, the image contains a large amount of related noise, causes considerable interference for target detection, causes the target detection accuracy to be low, and easily misses detection and false detection [20].

After the introduction of a convolutional neural network (CNN) [21], deep learning has been more successful in target detection. Regions with CNN features (R-CNN) can use convolutional neural networks to solve target-detection problems, creating the framework of Region Proposal + CNN [22]. However, due to a series of problems such as low efficiency and long time spent, R-CNN has not been widely used. Compared with R-CNN, Fast R-CNN uses shared convolution to reduce the overall network consumption and improve training and testing speeds [23]. However, Fast R-CNN uses selective search, which requires 2~3 s to extract candidate regions and only 0.32 s to extract features for classification [24], which does not satisfy the demand of real-time applications. Faster R-CNN combines the fully convolutional network and replaces the original selective search with a region proposal network (RPN) [25], which markedly speeds up training and detection.

In recent years, the YOLO (You Only Look Once) algorithm [26] has defined the target detection problem as an image-space border regression and class prediction problem. YOLO contains only a single network without target candidate region extraction, which markedly reduces calculation complexity, and although the detection accuracy is not as good as that of Faster R-CNN, its detection speed is 10 times faster than that of Faster R-CNN [27]. YOLOv2 [28] and YOLOv3 [29] changed some structures due to low detection accuracies. YOLOv2 uses the anchor boxes to predict the location of objects in an image, batch normalization in the convolutional layers, and a high-resolution classifier to improve accuracy. In YOLOv3, the previous backbone network of YOLOv2 (Darknet-19) was replaced with Darknet-53, binary cross entropy was used in loss calculations, and logistic regression was used to predict the “objectness score” for each bounding box [30].

Both methods are used in research in various areas of agriculture, such as detecting cattle’s faces to manage livestock [31], the use of YOLO to estimate fruit load [32], to classify date fruits by color perception [33], and to detect ungulate to prevent them from damaging crops [34]. Comparing the results of Faster R-CNN and YOLOv3, Faster R-CNN achieves higher accuracies, although the accuracy of this algorithm comes at the cost of time complexity [35].

This study described a solution for low-light conditions in orchards that allows an effective vision system to be used for vehicle navigation, which requires accurate target detection with lower machine operation speed. Therefore, Faster R-CNN was used for object detection in this study. In addition, the Faster R-CNN has the potential to unlock thermal imagery in agricultural orchard applications under low- and no-light conditions to increase working efficiency. Thermal cameras use infrared information to detect targets in an orchard. Therefore, the objective of this study was to use a thermal camera for tree-trunk detection as target objects using Faster R-CNN to develop an autonomous speed sprayer navigation system. In the first part of this article, the thermal camera accuracy is analyzed to confirm the accuracy of object detection under the different lighting conditions while considering distances at different orientations. The details of Faster R-CNN and preparation of the training dataset are explained in this section. In the second part, the calibration, validation, and testing results are shown and analyzed. The strengths and potential of the research are discussed in the Section 3. Finally, the fourth part provides conclusions and proposes topics for future research on navigation systems for autonomous speed sprayers.

## 2. Materials and Methods

### 2.1. Calibration of Thermal Camera

**Figure 1:**
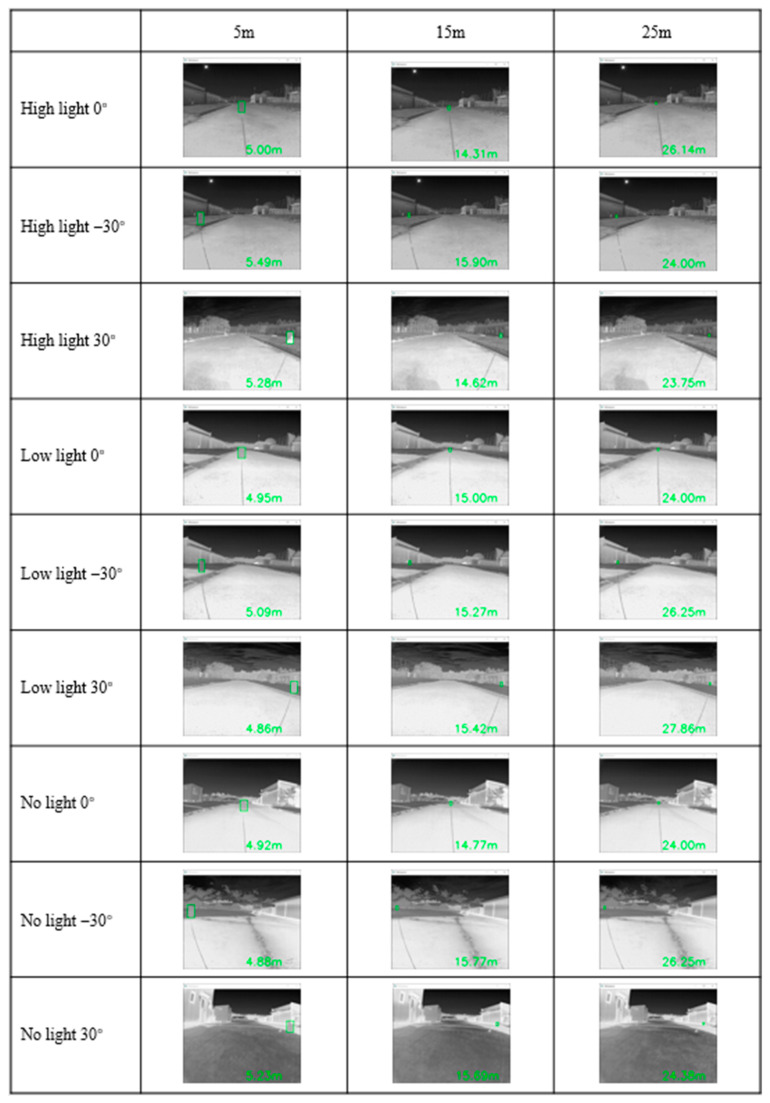
Object detection under different lighting conditions at a range of 25 m from the thermal camera with orientations of 0°, −30°, and 30° directions (green indicates the measured distance by camera).

A thermal camera (FLIR ADK^TM^, Teledyne FLIR LLC, Wilsonville, OR, USA) was used in each experiment, and calibration was performed to confirm the object detection performance under different lighting conditions while considering the orchard environment for different ranges (3, 5, 10, 15, 20, and 25 m) and orientations (0°, −30°, and 30° directions) of the thermal camera (Figure 1). The distance and orientations were measured using a laser distance meter (BOSCH GLM 250 VF).

Images were collected, and calibration was performed before each experiment to develop datasets from pear orchards. The experimental thermal camera was monocular and had a field of view (FOV) of 32°. The width and height of the target were required to be known to measure the distances. A 570 × 350 mm box was used as the target for confirmation of the accuracy of object detection by the thermal camera. According to the selected ranges and orientations, the returned distance and values were used to recognize the object in the image of the thermal camera.

### 2.2. Field Data Collection

**Figure 2:**
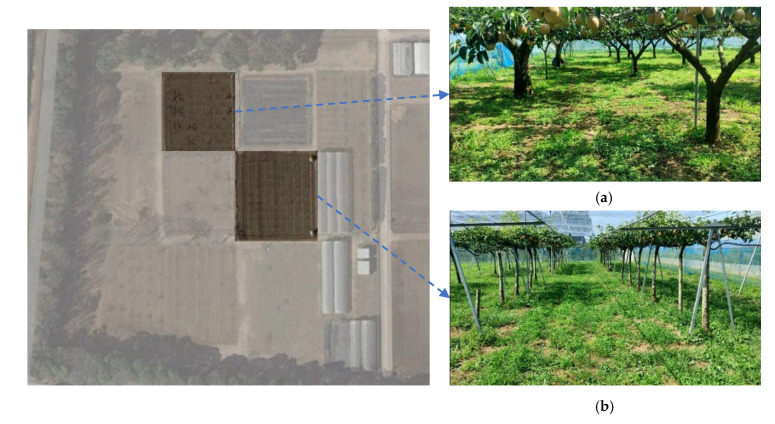
Aerial view of experimental pear orchards: (**a**) conventional planted pear orchard, and (**b**) joint-tree training pear orchard in the Tsukuba-Plant Innovation Research Center (T-PIRC), University of Tsukuba, Japan.

**Figure 3:**
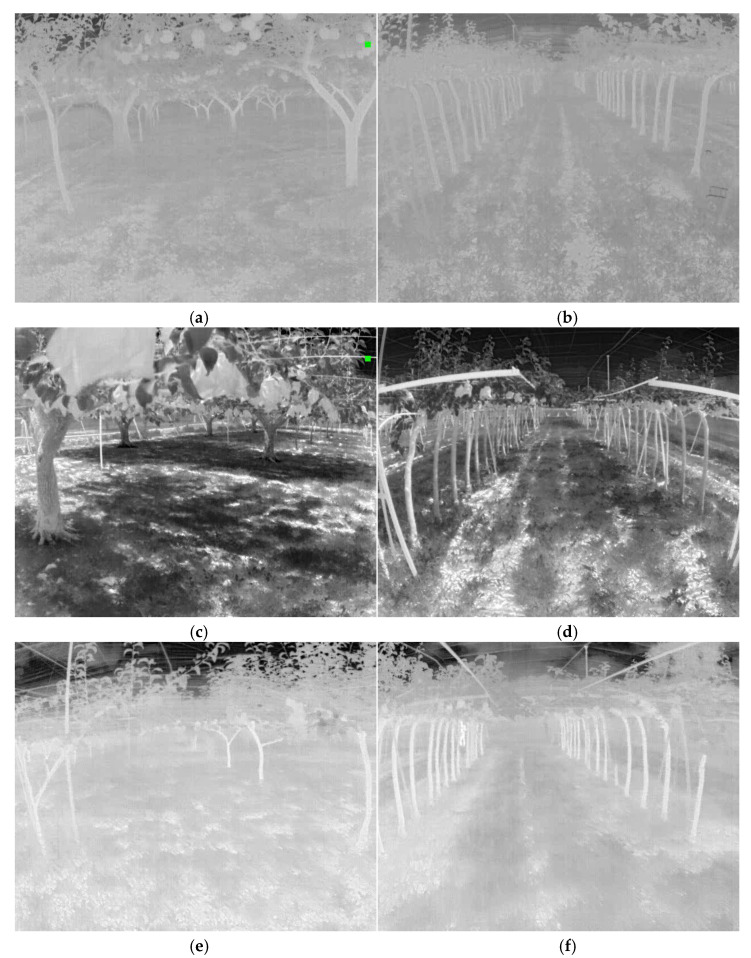
Analysis of thermal images under different lighting conditions: (**a**,**b**) no-light conditions (7–8 PM), (**c**,**d**) high-light conditions (12–2 PM), and (**e**,**f**) low-light conditions (5–6 PM).

The FLIR ADK^®^ thermal camera has a resolution of 640 × 512, and images were collected from pear orchards at the Tsukuba-Plant Innovation Research Center, University of Tsukuba, Tsukuba, Ibaraki (36°06′56.8″ N, 140°05′37.7″ E). In this study, two types of pear orchards were used for data collection: (1) a conventional pear orchard, and (2) a joint-tree training pear orchard (Figure 2). Images were collected three times under high-light (12–2 PM), low-light (5–6 PM), and no-light (7–8 PM) conditions in both orchards (Table 1). Thermal images under different light conditions, such as no-, high-, and low-light conditions, were then analyzed (Figure 3).

### 2.3. Data Preparation

#### 2.3.1. Image Frames from Videos

MATLAB^®^ (Matrix Laboratory, Natick, MA, USA) was used to capture images every 15 frames and remove images that were blurred and that did not contain tree trunks. In total, 5313 images were analyzed, and the number of images of trunks in different periods was similar.

#### 2.3.2. Labeling

LabelImg^®^ was used to label datasets while preparing visual object class (VOC) datasets. When the position of the tree trunk on the image was selected, information such as the image name, object classification, and pixel coordinates were recorded in an .xml file.

#### 2.3.3. Data Augmentation

To obtain a larger database and improve training accuracy, the original 5313 images were randomly flipped and rotated at random angles to obtain 7563 new images, and the labeling boxes of the images were also changed and saved as new .xml files.

#### 2.3.4. Data Splitting

After labeling was completed, the data were divided into three sets: training, validation, and testing. Training was conducted to adjust the model parameters, and the classifier was adjusted to increase accuracy. Validation was performed to check the state and convergence of the model developed during training, and validation sets were used to adjust hyperparameters and determine which set of hyperparameters was most suitable. Testing was performed to evaluate the generalizability of the model, which was used for the validation set to determine the parameters.

### 2.4. Training Model Development

#### 2.4.1. Faster R-CNN

**Figure 4:**
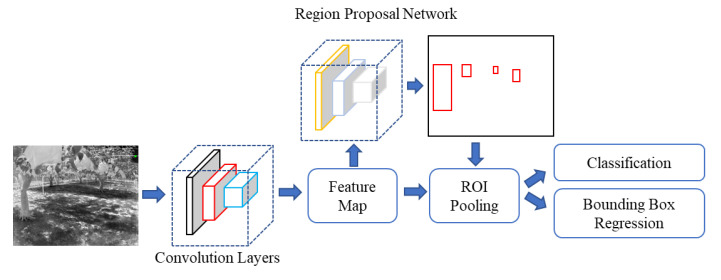
Faster R-CNN structure for tree truck detection used in this research.

**Figure 5:**
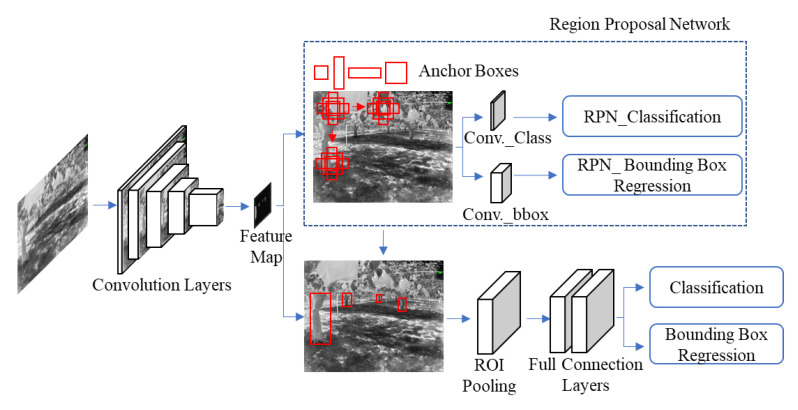
Faster R-CNN network structure-focusing regional proposal network for feature map.

In this study, the Faster R-CNN model was based on Fast R-CNN. Replacing the selective search in Fast R-CNN with RPN, to achieve more accurate regional proposals, reduce the redundancy of network computation, and improve detection speed. The structure of the Faster R-CNN model (Figure 4) and the network structure-selected images were kept for the datasets (Figure 5). The primary part of the model had convolutional layers, Regional Proposal Network (RPN), Region of Interest (ROI) pooling, and classification.

**Figure 6:**
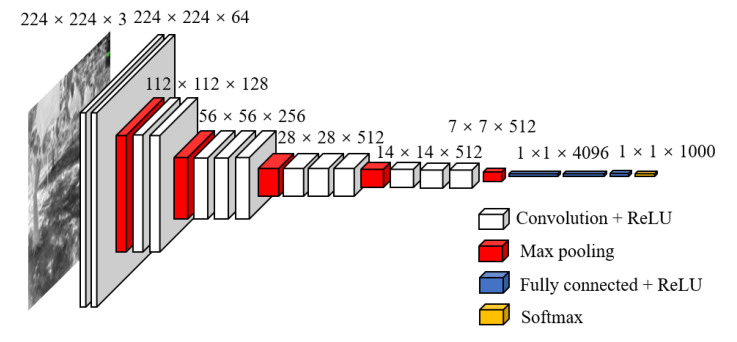
VGG16 for target of tree trunk detection.

(1) Convolutional layers: As a CNN network target detection method, Faster R-CNN first used a set of basic convolutional + relu + pooling layers to extract feature maps of images for training datasets. The feature maps were shared for subsequent RPN layers and fully connected layers. The feature extraction process in Faster R-CNN is the same as that in CNN and can be performed using some common structures, such as the commonly used Visual Geometry Group (VGG) and ResNet [36]. In this study, the VGG16 model was used, which is a convolutional neural network model that was proposed by Simonyan and Zisserman [37]. VGG can be divided into 6 configurations (A, A-LRN, B, C, D, E) according to the size of the convolutional kernel and the number of convolutional layers. This study used the D configuration with 13 convolutional layers, 3 fully connected layers, and 5 pooling layers. The convolutional layer and fully connected layer had weight coefficients called weight layers (Figure 6).

The convolutional layer was used to extract features. A 3 × 3 convolutional kernel was used to slide over the image, and the output changed the image size because both the stride and padding were 1. As the linear model could not solve all problems, the ReLU layer was used to add nonlinear factors to simulate more subtle changes [38]. The pooling layer compressed the input feature map to make the feature map smaller and simplify the computational complexity of the network, and feature compression was used to extract the primary features. The pooling layer used a 2 × 2 convolutional kernel with a stride of 2 to perform the max pooling in the feature map. Therefore, the output feature map size was reduced by half. Finally, a fully connected layer connected all of the feature maps and transmitted the output to the softmax classifier.

In this VGGNet, there were 5 convolutional segments, all of which used 3 × 3 convolutional kernels because the 3 × 3 convolutional kernels can have fewer parameters and have more nonlinear transformations to increase the CNN’s ability to learn features [37].

(2) RPN: This network model is a new structure proposed by Faster R-CNN, which determines the approximate position of the target from the feature maps. RPN first generated many anchors (the candidate boxes) on the feature map. Then, the RPN network was divided into two lines: the upper line distinguished whether it was a tree trunk by softmax classification, and the lower line was used to calculate the bounding box regression offset for the anchors to obtain an accurate proposal [39]. The final proposal layer combined the anchors of the tree trunk and the corresponding bounding box regression offsets to obtain the proposals and eliminate the proposals that were too small or out of bounds.

(3) ROI pooling: After the RPN network had created the proposals, ROI pooling was applied to the proposals, and the feature maps were generated by the last layer in the VGG16 network. In addition, a fixed-size proposal feature map was obtained and sent to localization and recognition.

(4) Classification: Softmax was used to classify the target objects of tree trunk detection. The output classification contained the probability of the target being a tree trunk. Bounding box regression offset each proposal and was used to predict the target detection box more accurately.

#### 2.4.2. Loss Function

The total loss function of Faster R-CNN consisted of classification loss and regression loss. The classification loss was calculated by softmax in the RPN classification layer, which was used to classify the anchors as positive and negative for training the network. Conversely, regression loss was calculated by the RPN bounding box regression layer and used to train the bounding box regression network. Therefore, the total loss function can be expressed as Equation (1):

(1) $$L(\left\{p_{i}\right\},\left\{t_{i}\right\}=\frac{1}{N_{cls}}\sum_{i}L_{cls}\left(p_{i},p_{i}^{*}\right)+\lambda \frac{1}{N_{reg}}\sum_{i}p_{i}^{*}L_{reg}\left(t_{i},t_{i}^{*}\right).\;$$

where *i* is the anchor index, *p_i_* represents the positive probability of anchor *I*, $p_{i}^{*}$ is the ground-truth label (if $p_{i}^{*}$ is 1, the anchor is positive; if $p_{i}^{*}$ is 0, the anchor is negative), *t* is the predicted bounding box, *t_i_* is the vector representing the 4 parameterized coordinates of the predicted bounding box, and $t_{i}^{*}$ is a positive anchor associated with the ground-truth box.

*N_cls_* is the trunk image during the training process, and *N_reg_* is the number of anchor locations. As the difference between *N_cls_* and *N_reg_* was too large in the real process, the parameter λ was used to balance the two parameters. Therefore, the network total loss calculation process was considered for 2 types of losses: *L_reg_* is the regression and *L_cls_* is the classification loss function for the detection of trunks. The smooth function (*L1*) was used to estimate regression loss (*L_reg_*), which is calculated and expressed as Equation (2):

(2) $$L_{reg}\left(t_{i},t_{i}^{*}\right)=\sum_{i\in \left\{x,y,w,h\right\}}smooth_{L1}\left(t_{i}-t_{i}^{*}\right).\;$$

The smooth function is defined as Equation (3):

(3) $$soomth_{L1}\left(x\right)=\{\begin{array}{c} 0.5x^{2}\frac{1}{\sigma^{2}}\;\;\;\;\;\left|x\right|\;\leq \;\frac{1}{\sigma^{2}}\\ \left|x\right|-0.5\;\;\;\;\;otherwise \end{array}.\;$$

where *x* is the prediction error of the bounding box and the parameter *σ* is used to control the smoothing area.

For the bounding box regression, we used the 4 coordinates in the following parameterized expressions (4)–(11):

(4) $$t_{x}=\frac{\left(x-x_{a}\right)}{w_{a}}\;.\;$$

(5) $$t_{y}=\frac{\left(y-y_{a}\right)}{h_{a}}.\;$$

(6) $$t_{w}=\log(w/w_{a}).\;$$

(7) $$t_{h}=\log(h/h_{a}).\;$$

(8) $$t_{x}^{*}=\frac{\left(x*-x_{a}\right)}{w_{a}}.\;$$

(9) $$t_{y}^{*}=\frac{\left(y*-y_{a}\right)}{h_{a}}\;.\;$$

(10) $$t_{w}^{*}=\log(w*/w_{a})\;.\;$$

(11) $$t_{h}^{*}=\log(h*/h_{a}).\;$$

where *x*, *y*, *w,* and *h* denote the center coordinates of the box and its width and height, respectively. The variables *x*, *x_a_*, and x* are used for the predicted box, the anchor box, and the ground truth box, respectively, which can be considered a bounding box regression from the anchor box to the nearby real box.

### 2.5. Training Platform and Validation

The hardware environment used in this study included Windows 10, Intel(R) Core (TM) i7-10750H CPU @ 2.60 GHz, 32.0 GB of RAM, and an NVIDIA GeForce RTX 2060. The software environment included Anaconda 3, Python 3.5, and TensorFlow-GPU 1.13.1. In the experiment, 12,876 infrared images of orchard trunks were used for training, 9270 images in the training datasets, and 2318 images in the validation datasets.

### 2.6. Model Testing 

To verify model reliability and stability, 1288 images were selected as the test set for validation after the model was trained. In this paper, the precision-recall curve was obtained by calculating precision and recall to highlight the trade-off between precision (P) and recall (R) of the classification model. The area under the curve was calculated to obtain the average precision (AP) of the model. As there was only one category in this experiment, AP was the same as mAP (mean average precision). The P, R, and AP can be expressed as expressions (12)–(14), respectively:

(12) $$P\;=\frac{TP}{TP+FP}\;.\;$$

(13) $$R\;=\frac{TP}{TP+FN}\;.\;$$

(14) $$\mathrm{AP}\;=\int_{0}^{1}PdR$$

where *TP* is the true positive value (i.e., the correct detection box); *FP* is the false-positive value (i.e., the false detection box that predicts the background as the target); and *FN* is the false negative value (i.e., the missed detection box). During testing, 1288 images were used as testing datasets in the Faster R-CNN model.

## 3. Results

### 3.1. Calibration Performance of Thermal Cameras in Different Lighting

**Figure 7:**
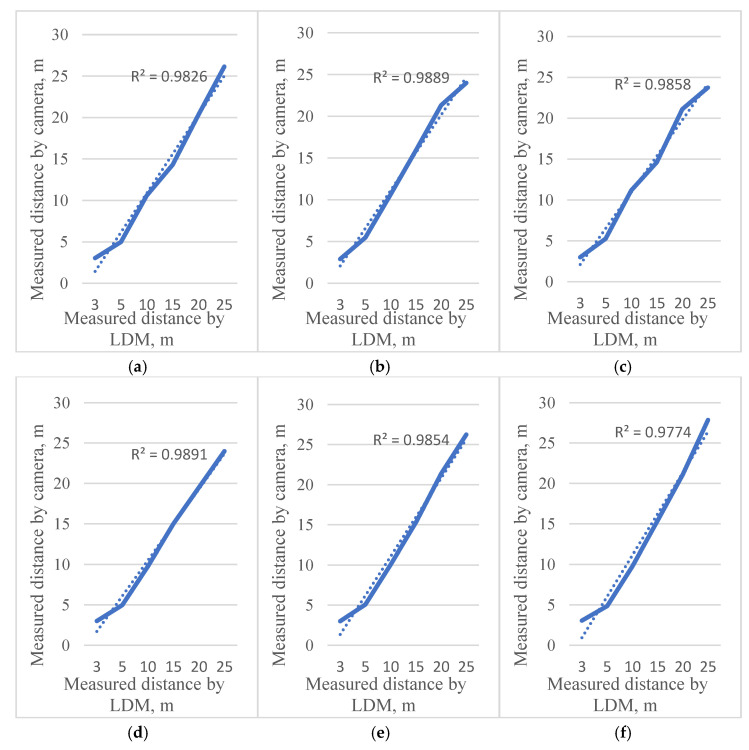

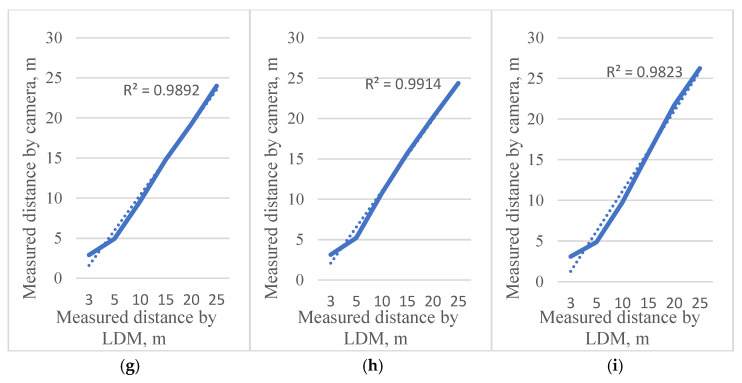
Measured distance under different lighting and orientations. (**a**–**c**) 0°, −30°, and 30° under high-light condition; (**d**–**f**) 0°, −30°, and 30° under low-light condition; (**g**–**i**) 0°, −30°, and 30° under no-light condition.

**Figure 8:**
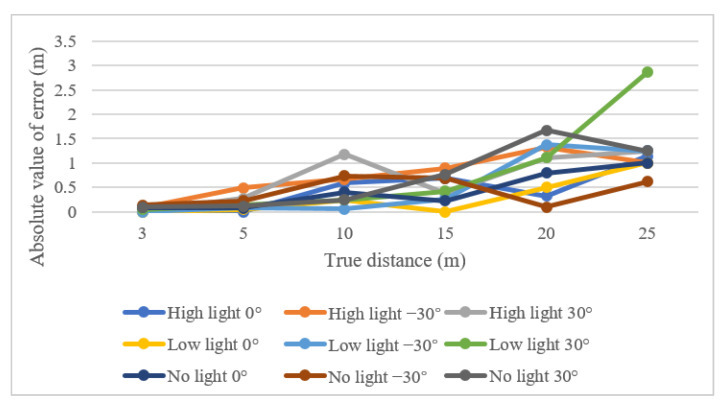
Measurement errors of target objects from a distance of 0 to 25 m under different lighting conditions and orientations of the thermal camera.

Under three light conditions, images at different angles and distances from the thermal camera were analyzed with a laser distance meter to confirm the camera ranges for object detection (Figure 7). The horizontal coordinate represents the real distance of the box, the vertical coordinate represents the distance value measured by the thermal camera, and R^2^ represents the residual sum of squares, which represents the error between the real distance and the measured distance. The average errors for distance measurement were 0.16 m for 5 m, 0.24 m for 15 m, and 0.3 m for 20 m. Under low-light conditions with the thermal camera oriented at 30°, the highest error of 2.86 m was observed to locate the target point from the range of 25 m from the camera. This measurement error occurred when the range of the distance was increased to recognize orchard tree trunk detection. At 20 m, the thermal camera error markedly increased. Tree trunk detection by thermal cameras at distances below 20 m was more satisfactory to implement in the positioning system of autonomous vehicles and the detection of objects (Figure 8).

### 3.2. Model Training and Validation

**Figure 9:**
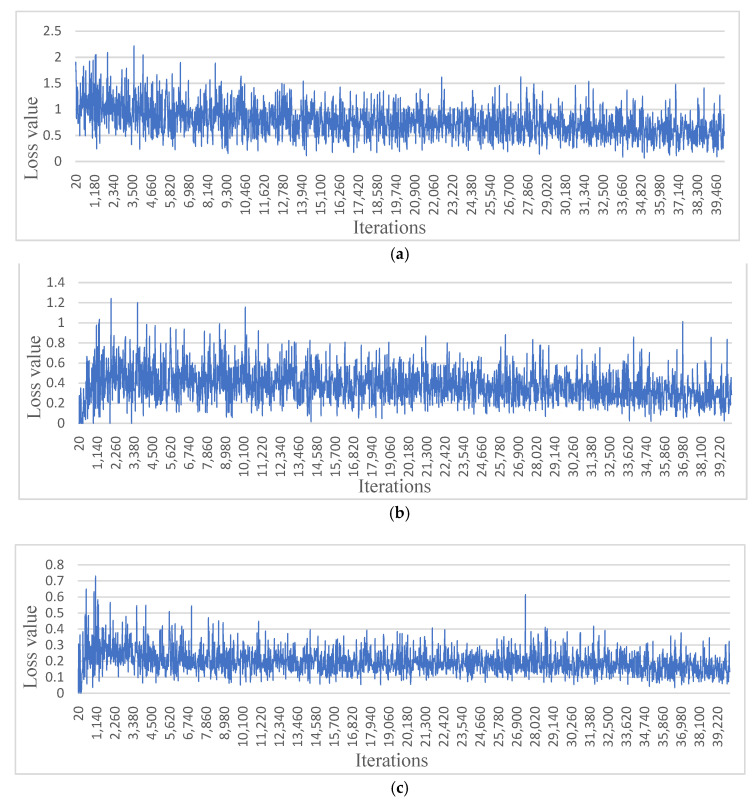

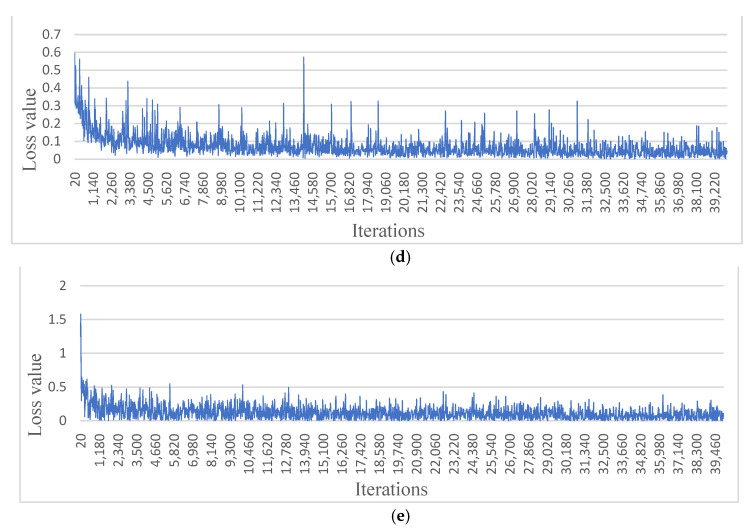
Faster R-CNN loss images. (**a**) Total loss. (**b**) Bounding box loss. (**c**) Classification loss. (**d**) Regional Proposal Network classification loss. (**e**) Regional Proposal Network bounding box loss.

**Figure 10:**
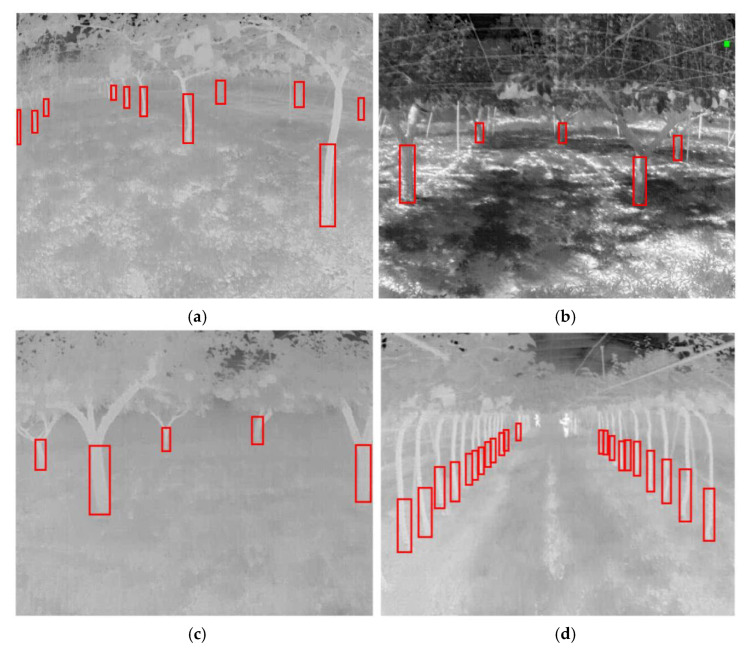

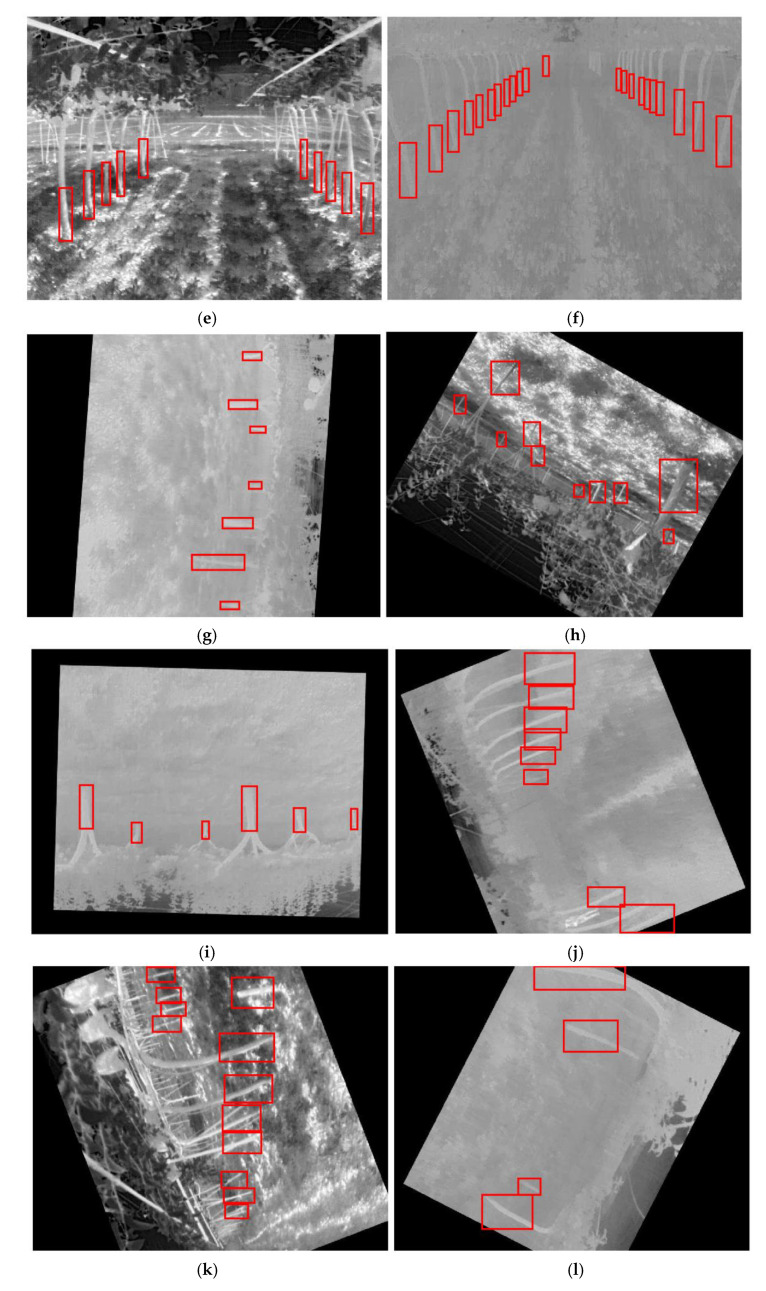
Validation results of Faster R-CNN. (**a**–**f**) Original images. (**g**–**l**) Randomly flipped and rotated images.

**Figure 11:**
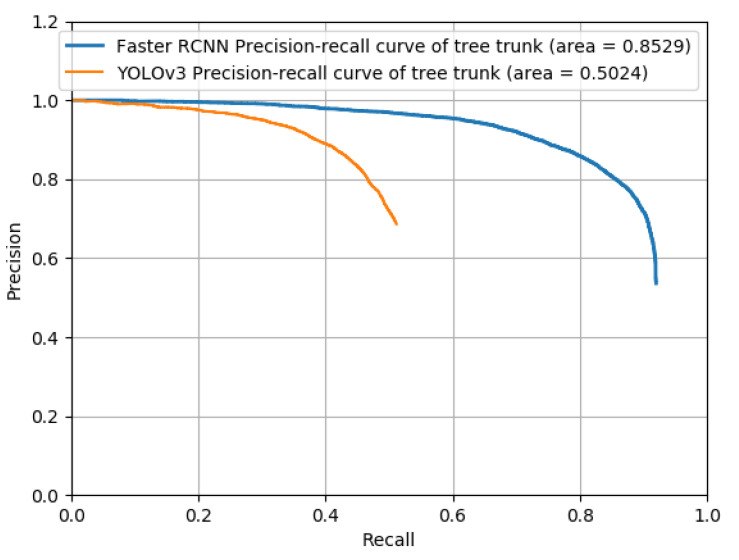
Precision-recall curve of Faster R-CNN and YOLOv3 validation.

The number of iterations were 40,000 (the training times are shown in Table 2), and the losses were reported as total loss, bounding box loss, classification loss, regional proposal network (RPN) classification loss, and RPN bounding box loss (Figure 9). According to Figure 9, the total loss function dropped to approximately 0.6 and oscillated near 0.6 when training reached 40,000 iterations, effectively reaching convergence. In addition, both the bounding box loss and classification loss converged; thus, training was stopped at 40,000 iterations. However, the results showed that the converged losses did not reach below 0.1, while other studies had less than 0.1 [40]; these results likely occurred because the two types of trees (conventional and joint) were investigated in this study, which differed markedly in shape. In addition, in the conventional orchard, the trees differed markedly in size, with some trees being 30 cm wide and some less than 5 cm, which led to a larger loss convergence result. In addition, randomly selected images were used to prepare the validation set. There were original images and randomly flipped and rotated images under the three lighting conditions (Figure 10). Compared with YOLOv3 using the same dataset and the same number of iterations, the mAP of YOLOv3 was 0.5024 and Faster R-CNN could reach 0.8529 (Figure 11). According to the validation results, the model worked properly to detect the tree trunk in conventional and joint orchards under high-, low-, and no-light conditions, and had a higher accuracy rate. The poles and shelves in the orchards could be distinguished from the fruit tree trunk. In the rotated images, the trunk could be identified accurately.

### 3.3. Model Testing

**Figure 12:**
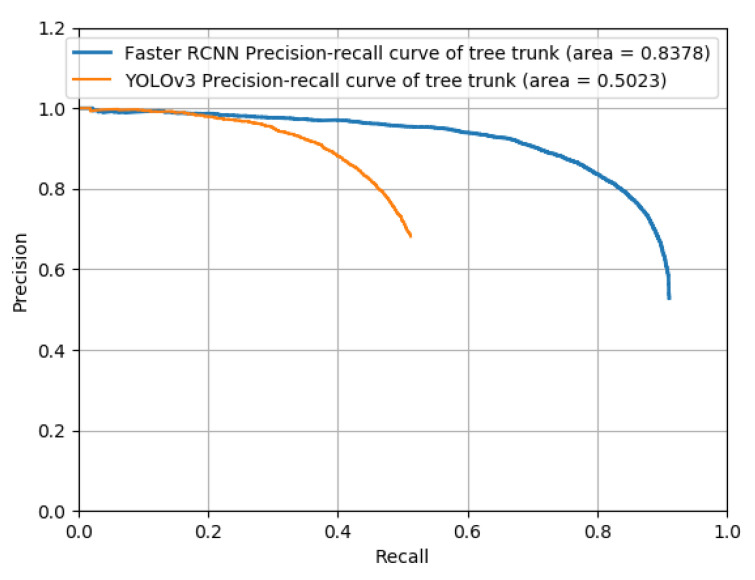
Precision-recall curve of Faster R-CNN and YOLOv3 testing.

**Figure 13:**
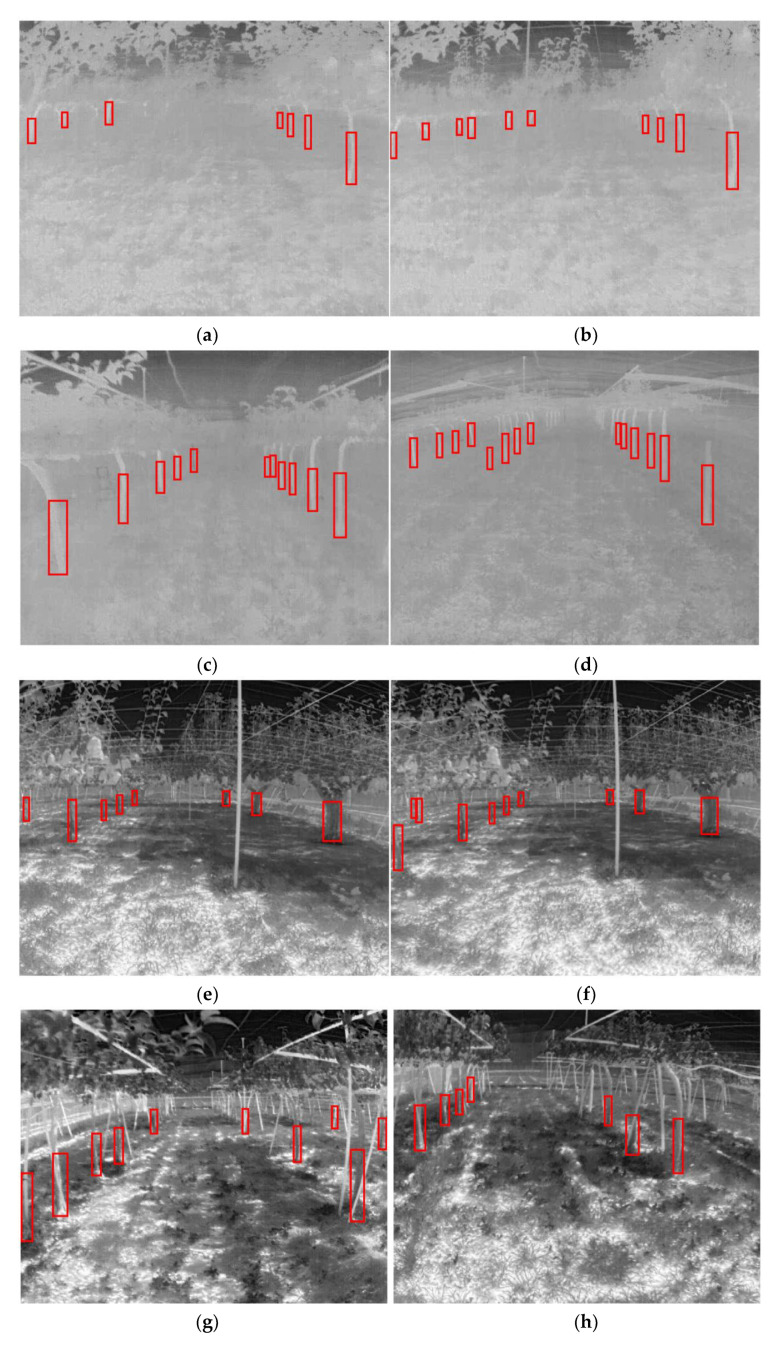

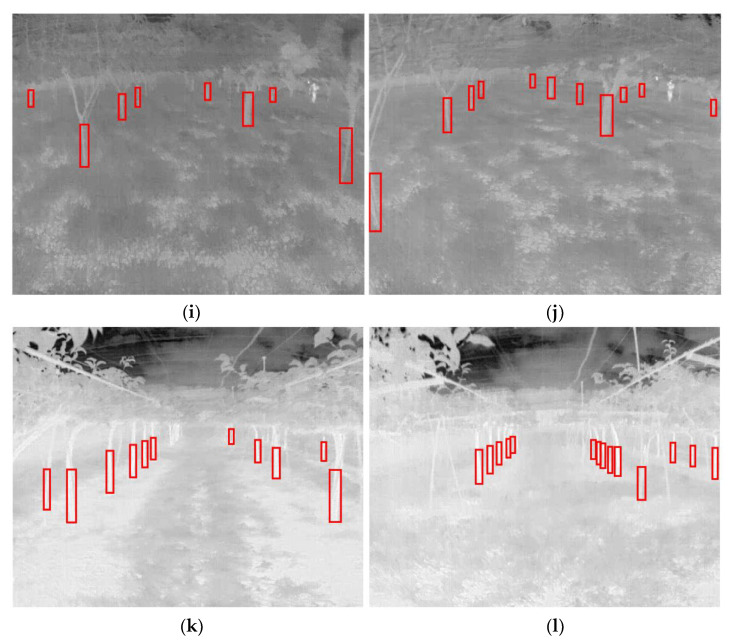
Image results of Faster R-CNN test: (**a**–**d**) no-light conditions, (**e**–**h**) high-light conditions, and (**i**–**l**) low-light conditions.

The mAP of the Faster R-CNN model was found to be 0.8378 (Figure 12). The images for testing were considered based on the training results conducted for the high-, low-, and no-light conditions (Figure 13). Two images were selected at different time periods, and the detection speed was related to the number of object proposals. In this study, we used 300 object proposals, and the detection speed was 83 ms in images and 90 ms in videos (Table 3). In the testing results in the conventional and joint orchards, this model was able to recognize tree trunks accurately. At longer distances of more than 20 m, image clarity decreased, and some errors in labeling the image occurred, which resulted in some omission and errors in the testing results for the recognition objects.

## 4. Discussion

Machine vision typically analyzes RGB imagery and thus cannot manage low-light conditions to recognize natural landmarks. Conversely, GNSS suffers signal interruptions due to dense canopies inside the orchards. LiDAR also has difficulties recognizing tree trunks because the scanning range decreases the chance of recognizing tree trunks [5]. To increase the robustness of these methods, this study used a thermal camera combined with deep learning to identify fruit tree trunks under high-, low-, and no-light conditions. The loss function shown in the Section 3 represents the difference between the predicted value of the model and the training samples. A smaller value indicates that the closer the predicted sample is to the real sample, the more robust the model is. In contrast, a larger value indicates that the difference between the predicted and real samples is larger [41]. The loss function in this research oscillated at approximately 0.6 due to the large variation in the shape and size of the trunks. According to the results, the accuracy of the Faster R-CNN testing was 83.8%. Compared with YOLO v3, Faster R-CNN had higher accuracy in the thermal image of this research. The model could distinguish poles and shelves of the same size, indicating that the thermal camera can be used for orchard navigation to detect tree trunks under different light conditions, and using this system can allow machinery to operate in orchards at any time. However, due to the unavoidable error in labeling, the recognition accuracies decreased to 20 m after the thermal camera and yielded some omissions and errors. In addition, for the thermal camera in monocular mode, distance measuring must be assisted by other sensors. The accuracy of the deep learning model can be improved with more experimental datasets that describe other lighting conditions and by improving the training process.

With comparison to our research outcomes, another contribution in tree trunk detection for forestry was conducted using different cameras (visible and thermal) to capture forest images [16]. Several deep learning-based nets were used to compare the accuracy and speed of detection through training, validation, and testing processes. The visible and thermal cameras can be used with mainly YOLO tiny and SSD for in-field forestry operations. In the results, visible imagery had a higher precision than thermal imagery. In forestry application, tree trunk detection can be helpful for mobile applications of robots; however, this research needs further extension to detect tree trunks at the different lighting and shadow stages, which was not discussed. In contrast, the presented research used one thermal camera to capture orchard images at the different times, demonstrated that thermal camera can be used in various light conditions through deep learning, and provided the basis for orchard navigation system. In the orchard navigation, more challenges exist with poles and the shape of trees. Our research considered different illumination times, poles, and included conventional pear orchard, and joint-tree training pear orchard together.

Therefore, in the joint tree system, the thermal camera has more flexibility to detect the tree trunk due to the uniform shape of the tree and growth in rows. However, natural orchards have different types, and the shapes of the field and canopy coverages can be markedly different. Traditionally, orchard growers in Japan control pruning and training according to their heights of operations, which makes using machinery inconvenient. Joint tree training systems have the advantage of uniform growing and machinery automation systems. Therefore, the thermal camera used in the research has a high application potential in joint tree training systems to use for orchard navigation in combination with other positional sensors such as LiDAR. In previous studies, LiDAR was able to detect artificial landmarks and cones for use in positional algorithms in orchard navigation [42,43]. In future research, a thermal camera is planned to be used to navigate speed sprayers in an orchard.

## 5. Conclusions

Thermal imagery has the potential to detect natural landmarks under different lighting conditions. This study proposed using the Faster R-CNN for thermal images to detect pear tree trunks to enable navigation under various lighting conditions in orchards. Conventional and joint tree training system orchards were considered for dataset collection. The accuracy and reliability of the model were verified by calibrating, training, and testing the thermal images, indicating that target detection could be performed under high-, low-, and no-light conditions using a thermal camera. In this study, all 640 × 512-pixel images and videos were used; 300 object proposals were generated for each image and frame; and this model achieved a mAP of 0.8378 in 83 ms with images and in 90 ms with videos. Faster R-CNN had higher accuracy compared to YOLO v3 using the same dataset and trained the same times, and speed sprayers typically use a lower speed (e.g., 3 m/s) in orchards to reduce the drift of pesticides and effective penetration of chemicals inside canopies. Therefore, the thermal camera can be used under high-, low-, or no-light conditions inside orchards to detect tree trunks using Faster R-CNN. In future research, an autonomous speed sprayer is planned to be used with a thermal camera and in combination with LiDAR for orchard navigation under different lighting conditions to provide solutions or orchard automation to increase productivity.

## Figures and Tables

**Table 1:** Dataset collection times and light conditions in orchards.

Date	Time	Light Condition
24 August 2021	19:00–20:00	No light
26 August 2021	13:00–14:00	Strong light
6 September 2021	17:00–18:00	Low light

**Table 2:** Iterations and training time of the deep learning model using Faster R-CNN.

Iterations Times	10,000	20,000	30,000	40,000
Training time	3.05 h	6.02 h	8.98 h	11.97 h

**Table 3:** Tree trunk detection time using the deep learning model of Faster R-CNN.

Objects Proposals	FPS	Detection Time (Images, ms)	Detection Time (Videos, ms)
100	13.9	72	72
200	12.3	81	81
300	11.1	83	90
400	10.6	93	95
500	9.9	95	101

## Data Availability

The dataset generated and analyzed during this study are available from the corresponding author upon reasonable request, but restrictions apply to the data reproducibility and commercially confident details.
